# Barriers to bowel scope (flexible sigmoidoscopy) screening: a comparison of non-responders, active decliners and non-attenders

**DOI:** 10.1186/s12889-018-6071-8

**Published:** 2018-10-05

**Authors:** Christian von Wagner, Bernardette Bonello, Sandro Stoffel, Hanna Skrobanski, Madeleine Freeman, Robert S Kerrison, Lesley M McGregor

**Affiliations:** 1 Research Department of Behavioural Science and Health, University College London, Gower Street, London, WC1E 6BT UK; 2 MRC/CSO Social and Public Health Sciences Unit, University of Glasgow, Glasgow, G2 3QB UK; 3 School of Health Sciences, University of Surrey, Guildford, GU2 7XH UK

**Keywords:** Bowel scope, Screening, Colorectal cancer, Flexible sigmoidoscopy, Non-attendance

## Abstract

**Background:**

Participation in bowel scope screening (BSS) is low (43%), limiting its potential to reduce colorectal cancer (CRC) incidence and mortality. This study aimed to quantify the prevalence of barriers to BSS and examine the extent to which these barriers differed according to non-participant profiles: *non-responders* to the BSS invitation, *active decliners* of the invitation, and *non-attenders* of confirmed appointments.

**Methods:**

Individuals invited for BSS between March 2013 and December 2015, across 28 General Practices in England, were sent a questionnaire. Questions measured initial interest in BSS, engagement with the information booklet, BSS participation, and, where applicable, reasons for BSS non-attendance. Chi-square tests of independence were performed to examine the relationship between barriers, non-participant groups and socio-demographic variables.

**Results:**

1478 (45.8%) questionnaires were returned for analysis: 1230 (83.2%) attended screening, 114 (7.7%) were non-responders to the BSS invitation, 100 (6.8%) were active decliners, and 34 (2.3%) were non-attenders. Non-responders were less likely to have read the whole information booklet than active decliners (*x*^2^ (2, *N* = 157) = 7.00, *p* = 0.008) and non-attenders (*x*^2^ (2, *N* = 101) = 8.07, *p* = 0.005). Non-responders also had lower initial interest in having BSS than either active decliners (*x*^2^ (2, *N* = 213) = 6.07, *p* = 0.014) or non-attenders (x^2^ (2, *N* = 146) = 32.93, *p* < 0.001). Overall, anticipated pain (33%) and embarrassment (30%) were the most commonly cited barriers to BSS participation. For non-attenders, however, practical, appointment-related reasons were most common (27%).

**Conclusions:**

Interventions to improve BSS uptake should be more nuanced and use targeted strategies to address the specific needs of each group.

## Background

Colorectal cancer (CRC) is the second most common cause of cancer-related deaths in the UK [1], making prevention and early diagnosis a priority in cancer control. Results from the UK Flexible Sigmoidoscopy (FS) Trial showed that a single FS examination with removal of pre-malignant growths reduced CRC mortality by 43% and CRC incidence by a third [2]. In response to these results, and other worldwide research supporting FS as a screening modality [3, 4], NHS England now offer men and women a single, free FS screen at age 55 as part of the NHS Bowel Cancer Screening Programme. This is known as ‘bowel scope screening’ (BSS) and was introduced in March 2013. A recent follow-up of the UK trial has shown that the benefits of a FS screen are retained 17 years after the initial examination [5], providing further evidence as to the need to increase uptake of the test. Uptake among the screening-eligible population will be key to realizing the projected public health benefits of this screening test [6].

A recent study analysing BSS invitations sent within the first 14 months of BSS roll out in England (21,187 invitations) found that only 43% of those invited attended their pre-booked appointment [7]. Furthermore, people from more socioeconomically deprived and ethnically diverse backgrounds were significantly less likely to take part and, in contrast to CRC screening using the home-based guaiac faecal occult blood test (gFOBt), women were significantly less likely to attend BSS than men (42% vs. 45%) [7]. For women, this highlights an almost twofold gap between participation in BSS and both breast and cervical screening [8, 9].

A recent qualitative study conducted with BSS invitees identified a number of practical and psychological barriers to attendance, and concluded that it was not the presence of concerns about the test but rather the strength of these concerns that was most important in making the decision to participate [10].

While important and informative, previous research into the barriers and facilitators of screening uptake has commonly considered non-participants as a single group; however, there is evidence to suggest that there are distinct sub-groups of non-participants. For example, in the UK FS trial, those who never returned the pre-trial questionnaire were less likely to remember receiving invitation materials and more likely to report procrastination than those who made an active decision not to participate [11]. Furthermore, a quantitative analysis of data from the UK FS trial demonstrated that non-intenders, non-attenders and attenders all had unique demographic and psychological profiles. Non-attenders were the most difficult group to characterise, with only 50% correctly classified by discriminant analyses [12]. A more nuanced understanding of non-participants is particularly important in trying to better understand to what extent decisions about cancer screening are based on well-informed choices. Previous research has identified distinct types of non-attenders within the context of breast cancer screening, and demonstrated among other socioeconomic and psychological differences that passive non-participants are more likely to come from socioeconomically deprived background than active non-participants [13, 14]. In a more a recent study, Marlow and colleagues highlighted that within non-participants of cervical screening, a majority had not made an active decision to not participate but rather lacked awareness or the means to translate their intention to attend into action [15].

In the context of the BSS, non-participants can be classified into three major groups: those who never respond to the invitation (non-responders, NRs), those who, following the receipt of the invitation or the reminder letter, choose to initiate contact with the screening centre in order to decline the offer (active decliners, ADs), and those who initially confirm but subsequently fail to attend their appointment (non-attenders, NAs). Understanding differences across these major groups of screening non-participants will help focus future research to address the concerns specific to each group. The aim of this study was to quantify the prevalence and combination of barriers to BSS and investigate the extent to which barriers vary across the different types of non-participants.

## Methods

### Sample population

Between May and October 2015, 28 General Practices located across Surrey, London, Norfolk, Tyne and Wear, and Wolverhampton were recruited to this study. Questionnaires were sent to registered patients within each practice who had received their BSS invitation within the last 2 years. Age was used as a proxy for this eligibility criteria; patients who were aged between 55 (+ 2 months) and 57 (+ 0 months) years were invited to participate. Individuals noted as not being able to read English were identified within each practice and excluded from the invitation process.

### Data collection

Each eligible individual was assigned a unique study ID number. The name, address and study ID number of each person was then sent to Docmail, an online hybrid mailing company, for the delivery of study packs. Each study pack contained a cover letter from the individual’s GP, an 8-page questionnaire booklet, and a freepost return envelope (addressed to University College London, UCL). The cover letter included an introduction to the study and instructions on how to participate, encouraging the return of the questionnaire (completed or not) within 2 weeks to avoid a reminder. Survey respondents were informed that by returning a completed questionnaire they were providing consent for their data to be used in this study.

All 28 practices sent a reminder to those who failed to return their questionnaire after an average of 4 weeks and included a replication of the original study pack. All but one practice sent a second reminder, containing a letter only, to those who had not returned a questionnaire after a further 4 weeks (on average). A unique study ID number was attached to each questionnaire and was used to identify those requiring a reminder; questionnaires were anonymous.

### Measures

For this study we focused our analysis on a selection of questions within the questionnaire that were relevant to our current research aim.

#### Demographic characteristics

Survey respondents were asked to select their response for gender (*Male; Female*), living arrangement (*Single; Married; Cohabiting/ living with partner; Divorced/ separated; Widowed*), ethnicity (*White; Other*), employment status (*Employed full-time; Employed part-time; Unemployed; Full-time homemaker; Retired; Student; Disabled or too ill to work; Self-employed*) and self-rated health (*Excellent; Good; Fair; Poor*). Age (in years) was requested as an open response.

#### Socioeconomic deprivation

An additional three demographic questions were asked in order to calculate a proxy measure for socioeconomic deprivation. One point was given to an individual if their household did not have a car or van, if they had no formal qualifications and if they did not own their own home [16, 17]. Scores, therefore, ranged from 0 to 3, with high scores indicating higher levels of social deprivation.

#### Index of multiple deprivation

To compare the socioeconomic status of responders and non-responders, practices provided a score on the Index of Multiple Deprivation (IMD) [18] for each patient they had invited. IMD is an area-based measure of deprivation based on income, employment, health and disability, education, skills and training, barriers to housing and services, crime and living environment, and can be identified from a postcode [18]. IMD scores are divided into quintiles, with quintiles 1 to 5 representing the least to the most deprived areas.

#### Interest in BSS

Participants who indicated they had received an appointment letter for BSS were asked: “*When you received the appointment letter, how interested were you in having the bowel scope screening test?*” Response options were: “*Very*”, “*Moderately*”, “*Slightly*”, and “*Not at all*”.

#### Engagement

The extent to which survey respondents were engaged with the information booklet sent with the BSS invitation was measured by asking: “*How much of the information booklet did you read?*”. Response options were: “*None of it*”, “*Some of it*”, “*Most of it*”, “*All of it*” and “*Don’t know*”.

#### BSS invitation response

Survey respondents were asked “*Did you respond to your appointment letter* (i.e. *confirm or cancel*)?”, with the following response options: “*Yes, I confirmed my appointment*”, “*Yes, I changed my appointment date or time*”, “*Yes, I cancelled my appointment*” and “*No, I did not respond*”. Those who selected “*No, I did not respond*”, were then asked if they had then received a reminder letter and, if so, how they had responded to that (response options were the same as above).

#### Self-reported BSS participation

To determine whether survey respondents had attended their BSS appointment and received a BSS test, the following question was asked: “*Did you have a bowel scope screening test?*”. Response options were “*Yes*” and “*No*”. To allow verification of BSS participation status, survey respondents were also asked at the end of the questionnaire if they wished to give permission for the research team to contact the NHS Bowel Cancer Screening Programme to check their response to the BSS invitation. Those who agreed were then asked to provide their name, date of birth and postcode.

#### Barriers to BSS

Survey respondents who stated that they had not participated in BSS were asked to indicate their reasons for non-attendance from a pre-specified list of 15 options. Survey respondents were instructed to select all reasons they deemed relevant to them (see Supplementary data page 3). Space was additionally provided for non-listed barriers to be added in an open text format. These were coded and included in the overall list of barriers for analysis.

### Statistical analysis

Chi-square tests of independence were performed to examine the relationship between defined participation/non-participation groups and socio-demographic variables (Tables 1 and 2). We used Fisher’s exact test (two-tailed, FET) for variables that had at least one cell with a frequency of less than 5, i.e. ‘deprivation’ and ‘interest’ (Table 2). Chi-square test of independence and Fisher’s exact test (two tailed) were also used, where appropriate, to examine the relationship between non-participation groups and single barriers^1^ (Tables 3 and 4). Where a test indicated a significant relationship, we conducted further pair-wise comparisons using Bonferroni adjusted alpha levels for the number of potential comparisons per variable (i.e. 0.05/3 = 0.016). We used Stata/IC version 14.1 (StataCorp LP, College Station, TX) to conduct the data analysis and only report significant results.

**Table 1:** Descriptive statistics of the overall study sample (row percentages)^a^

	Participants (*N* = 1079)		Non-participants (*N* = 243)		*p*-value^*^
	*N*	(%)	*N*	(%)	
Age					
55 years	254	(23.54)	53	(21.81)	0.385
56 years	667	(61.82)	146	(60.08)	
57 years	158	(14.64)	44	(18.11)	
Gender					
Male	528	(48.93)	103	(42.39)	0.065
Female	551	(51.07)	140	(57.61)	
Ethnicity					
Other	75	(6.97)	27	(11.20)	0.026
White	1001	(93.03)	214	(88.80)	
Living arrangement					
Married or living with partner	860	(79.85)	179	(73.97)	0.043
Single, divorced or widowed	217	(20.15)	63	(26.03)	
Markers of socioeconomic deprivation					
markers	845	(78.97)	170	(70.54)	< 0.001
1 marker	171	(15.98)	42	(17.43)	
2–3 markers	54	(5.05)	29	(12.03)	
Index of Multiple Deprivation IMD					
1st quintile (least deprived)	401	(37.72)	80	(33.76)	0.239
2nd quintile	262	(24.65)	53	(22.36)	
3rd quintile	177	(16.65)	40	(16.88)	
4rd quintile	130	(12.23)	33	(13.92)	
5th quintile (most deprived)	93	(8.75)	31	(13.08)	
Paid employment					
No	172	(16.09)	61	(25.31)	0.001
Yes	897	(83.91)	180	(74.69)	
Self-rated health					
Excellent, good	932	(86.46)	179	(73.97)	< 0.001
Fair, poor	146	(13.54)	63	(26.03)	
Initial interest in Bowel Scope					
Very, moderately	1022	(95.13)	123	(51.04)	< 0.001
Slightly, not at all	52	(4.87)	118	(48.96)	
Extend to which book was read					
None, some or most	252	(28.00)	78	(44.32)	< 0.001
All of it	648	(72.00)	98	(55.68)	

Note that missing cases are not reported, so that the column frequencies do not always sum up to the total stated at the top of the table

^a^Only eligible sample (i.e. aged between 55 and 57 years)

^*^The *p*-values are derived from Chi-square tests of Independence

**Table 2:** Predictors of screening participation (complete case analysis for both unadjusted and adjusted models, *N* = 1036)

	(%)	Unadjusted		Adjusted	
		Odds ratio	95% CI	Odds ratio	95% CI
Age					
55 years	82.9%	Ref		Ref.	
56 years	84.4%	1.121	0.760–1.656	1.072	0.667–1.721
57 years	82.0%	0.941	0.555–1.596	0.836	0.443–1.577
Gender					
Male	86.0%	Ref		Ref.	
Female	81.7%	0.729	0.522–1.019	0.770	0.512–1.157
Ethnicity					
Other	74.0%	Ref		Ref.	
White	84.5%	1.910	1.115–3.272^*^	1.065	0.524–2.162
Living arrangement					
Married/living with somebody	85.0%	Ref		Ref.	
Single/div./wind.	78.8%	0.657	0.450–0.959^*^	0.752	0.463–1.222
Index of Multiple Deprivation IMD					
1st quintile	85.2%	Ref		Ref.	
2nd quintile	85.0%	0.980	0.629–1.526	1.005	0.593–1.700
3rd quintile	84.0%	0.911	0.554–1.497	0.935	0.513–1.706
4rd quintile	80.0%	0.693	0.412–1.164	0.807	0.419–1.555
5th quintile	78.4%	0.627	0.359–1.095	1.082	0.518–2.259
Markers of socioeconomic deprivation					
0 markers	85.1%	Ref		Ref.	
1 marker	83.0%	0.858	0.547–1.346	1.416	0.796–2.517
2–3 markers	67.2%	0.359	0.204–0.635^**^	0.807	0.371–1.755
Paid employment					
No	76.5%	Ref		Ref.	
Yes	85.3%	1.785	1.211–2.629^**^	1.407	0.856–2.311
Self-reported health					
Excellent/good	69.8%	Ref		Ref.	
Fair/poor	86.3%	2.728	1.853–4.016^**^	2.306	1.410–3.770^**^
Initial interest in Bowel Scope					
Very/moderately	30.3%	Ref		Ref.	
Slightly/not at all	90.8%	22.727	14.542–35.519^**^	22.292	13.768–36.094^**^
Extend to which booklet was read					
None/some of it	76.5%				
All of it	86.8%	2.027	1.445–2.842^**^	1.136	0.737–1.751
*N*		1036		1036	

^*^ *p* < 0.05; ^**^ *p* < 0.01

**Table 3:** Descriptive statistics of the three non-participant groups (column percentages)^a^

	NRs (*N* = 110)		ADs (*N* = 99)		NAs (*N* = 34)		*p*-value^*^
	*N*	(%)	*N*	(%)	*N*	(%)	
Age							
55 years	28	(25.45)	19	(19.19)	6	(17.65)	0.666
56 years	61	(55.45)	64	(64.65)	21	(61.76)	
57 years	21	(19.10)	16	(16.16)	7	(20.59)	
Gender							
Male	57	(51.82)	34	(34.34)	12	(35.29)	0.026
Female	53	(48.18)	65	(65.66)	22	(64.71)	
Ethnicity							
Other	10	(9.17)	11	(11.22)	6	(17.65)	0.393
White	99	(90.83)	87	(88.78)	28	(82.35)	
Living arrangement							
Married or living with partner	76	(69.72)	78	(78.79)	25	(73.53)	0.330
Single, divorced or widowed	33	(30.28)	21	(21.21)	9	(26.47)	
Markers of socioeconomic deprivation							
0 markers	62	(56.88)	82	(83.67)	26	(76.47)	0.001^a^
1 marker	26	(23.85)	11	(11.22)	5	(14.71)	
2–3 markers	21	(19.27)	5	(5.10)	3	(8.82)	
Paid employment							
No	26	(23.85)	22	(22.45)	13	(38.24)	0.169
Yes	83	(76.15)	76	(77.55)	21	(61.76)	
Self-rated health							
Excellent, good	81	(74.31)	71	(71.72)	27	(79.41)	0.674
Fair, poor	28	(25.69)	28	(28.28)	7	(20.59)	
Initial interest in Bowel Scope							
Very, moderately	39	(35.78)	53	(53.54)	31	(93.94)	< 0.001^a^
Slightly, not at all	70	(64.22)	46	(46.46)	2	(6.06)	
Extent to which book was read							
None, some or most	45	(59.21)	28	(35.90)	5	(22.73)	0.001
All of it	31	(40.79)	50	(64.10)	17	(77.27)	

*NRs* Non Responders, *ADs* Active Decliners, *NAs* Non-Attenders

^a^Only eligible sample who did not attend the screening (i.e. aged between 55 and 57 years)

^*^The *p*-values are derived from Chi-square tests of Independence, except for ‘markers of socioeconomic deprivation’ and ‘initial interest in bowel scope’ which were both assessed with Fisher’s exact test^a^

**Table 4:** Number of reasons for not participating stated by non-attenders

	Total (*N* = 243)		NRs (*N* = 110)		ADs (*N* = 99)		NAs (*N* = 34)		*p*-value^*^
	*N*	(%)	*N*	(%)	*N*	(%)	*N*	(%)	
None	34	(13.99)	8	(7.27)	22	(22.22)	4	(11.76)	0.002
One	89	(36.63)	36	(32.73)	34	(34.34)	19	(55.88)	
Two or more	120	(49.38)	66	(60.00)	43	(43.43)	11	(32.35)	

*NRs* Non Responders, *ADs* Active Decliners, *NAs* Non-Attenders

^*^The *p*-value is derived from Fisher’s exact test

## Results

### Questionnaire response rate

The study questionnaire was sent to 3226 eligible individuals with 1478 (45.8%) completed questionnaires returned. A further 292 (9.1%) were returned blank. Using study ID numbers and data available in GP records, selected comparisons, i.e. gender and area level deprivation (using postcodes converted to Index of Multiple Deprivation scores), could be made between those who returned a completed questionnaire, those who returned a blank questionnaire (indicating they did not want to respond to the survey) and those who did not respond at all. Women were found to be significantly more likely to answer the questionnaire than men (48.1% vs 43.5%, *x*^2^ (1, *N* = 3226) = 6.78, *p* = 0.009). Furthermore, responders to the questionnaire were more likely to live in a less deprived area (*x*^2^ (4, *N* = 2915) = 13.30, *p* < 0.001).

**Fig. 1:**
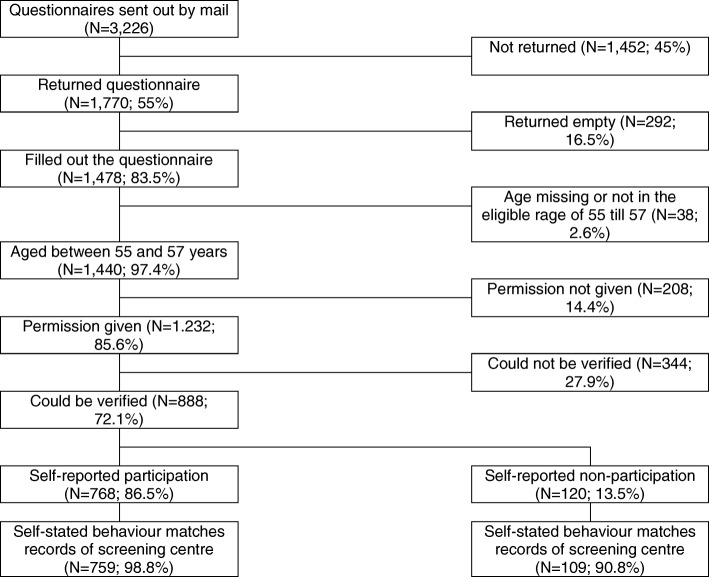
The flow of participants through the study

Among those who returned a completed questionnaire, 38 (2.6%) were removed from the analysis due to stated age being older or younger than expected for the study sample. Of the questionnaire respondents included in the final analysis (*n* = 1440), the majority were female (52.9%), married or cohabiting (78.2%), white (91.6%), and living in areas with the lowest levels of deprivation (38.8%). Figure 1 shows the flow of survey respondents through the study.

### BSS attendance

#### Variation in socio-demographic variables by BSS attendance status

Table 1 presents a summary of demographic variables by participation. Due to small sample size, response options were combined for some variables. Among those who returned a completed questionnaire, 1079 (81.62%) confirmed and attended their BSS appointment, and 243 (18.38%) did not. Among those who gave us permission, there was 90.8% correspondence between self-reported and verified participation status.

BSS non-participants were more likely to be from ethnically diverse backgrounds (*x*^2^ (2, *N* = 1317) = 4.94, *p* = 0.026), not living with a partner (*x*^2^ (2, *N* = 1319) = 4.09, *p* = 0.043), more deprived (*x*^2^ (2, *N* = 1311) = 8.00, *p* = 0.005), and in paid employment (x^2^ (2, *N* = 1310) = 11.44, *p* = 0.001). Similarly, those who did not participate in BSS were more likely to report a poor health status (*x*^2^ (2, *N* = 1320) = 23.13, *p* < 0.001), and low initial interest in having the BSS test (*x*^2^ (2, *N* = 1315) = 340.39, *p* < 0.001). Relatedly, BSS non-participants were also less likely to have read the whole information booklet (*x*^2^ (2, *N* = 1076) = 18.44, *p* < 0.001). A multivariable regression (see Table 2) demonstrated that only self-reported health and initial interest in bowel scope remained significant predictors of BSS attendance.

#### Variation in socio-demographic variables by non-participant subgroups

Of the 243 BSS non-participants, 110 did not respond to their BSS invitation (NRs; 45.3%), 99 actively declined their BSS invitation (ADs; 40.7%) and 34 confirmed their BSS appointment but subsequently failed to attend (NAs; 14.0%). Table 3 displays the socio-demographic variables for the three different types of non-participation. Chi-square tests of independence showed that the three types of non-participation differed in their gender composition and engagement with the information booklet (*p* < 0.05). Fisher’s exact test showed that initial interest in screening and socioeconomic deprivation also significantly varied across the three groups (*p* < 0.05, FET).

Pairwise comparisons, using a Bonferroni corrected significance level (*p* < 0.016), revealed that compared to ADs, NRs were more likely to be male (*x*^2^ (2, *N =* 209) = 6.47, *p* = 0.011) and be more deprived (*p* < 0.001, FET). NRs were less likely to have read the whole booklet than ADs (*x*^2^ (2, *N* = 154) = 8.39, *p* = 0.004) or NAs (*x*^2^ (2, *N* = 98) = 9.09, *p* = 0.003). Similarly, NRs stated significantly lower initial interest in having the BSS test than either ADs (*x*^2^ (2, *N* = 208) = 6.63, *p* = 0.010) or NAs (*p* < 0.001, FET). While there was no difference between ADs and NAs with regard to their level of engagement, ADs indicated significantly lower levels of initial interest in BSS (*p* < 0.001, FET).

### Barriers

#### Number of barriers

209 (86%) BSS non-participants gave at least one reason for not participating in BSS: NRs = 102 (93%), ADs = 77 (78%) and NAs = 30 (88%); Table 4. Those who answered the question gave on average 2.12 reasons (NRs = 2.35, ADs = 2.03 and NAs = 1.6). The number of reasons endorsed varied by the type of non-participant (*p* = 0.002, FET). Pairwise comparisons show that NRs were more likely to state two or more reasons for not participating than ADs (*x*^2^ (2, *N* = 209) = 10.89, *p* = 0.004) and NAs (*p* = 0.014, FET). There was no difference between ADs and NAs (*p* = 0.084, FET).

#### Barriers by subgroup

Table 5 shows the list of barriers and the proportion of the three non-participant groups endorsing them (the two items on the enema were combined). Worrying the test would be painful (33.01%), embarrassing (30.14%) and simply not personally needed (25.84%) were the three most commonly endorsed barriers overall. One interesting result was that NAs had a lower rate of “not needed” and a higher rate of “other test done”, While these two items could have been referring to a related issue. However, Appendices 1 and 2 show that none of our respondents ever endorsed both items together.

**Table 5:** Stated reasons for not participating across the three non-participant groups (*N* = 209)

	Total (*N* = 209)		NRs (*N* = 102)		ADs (*N* = 77)		NAs (*N* = 30)		*p*-value^*^
	*N*	%	*N*	%	*N*	%	*N*	%	
Worried about pain	69	(33.01)	38	(37.25)	25	(32.47)	6	(20.00)	0.208^b^
Embarrassing	63	(30.14)	39	(38.24)	22	(28.57)	2	(6.67)	0.002
Not needed	54	(25.84)	31	(30.39)	22	(28.57)	1	(3.33)	0.004
Worried about harm	53	(25.36)	23	(22.55)	25	(32.47)	5	(16.67)	0.159^b^
Appointment problems	51	(24.40)	24	(23.53)	19	(24.68)	8	(26.67)	0.938^b^
Enema not wanted	44	(21.05)	24	(23.53)	14	(18.18)	6	(20.00)	0.678^b^
No time	34	(16.27)	20	(19.61)	10	(12.99)	4	(13.33)	0.469
Worried about result	16	(7.66)	15	(14.71)	0	(0.00)	1	(3.33)	< 0.001
Medical reasons	16	(7.66)	4	(3.92)	5	(6.49)	7	(23.33)	0.005
Unacceptable	12	(5.74)	7	(6.86)	5	(6.49)	0	(0.00)	0.428
Bad experience	11	(5.26)	6	(5.88)	3	(3.90)	2	(6.67)	0.760
Other test done^a^	11	(5.26)	2	(1.96)	5	(6.49)	4	(13.33)	0.030
Forgot	7	(3.35)	6	(5.88)	0	(0.00)	1	(3.33)	0.070
Financial^a^ problems	3	(1.44)	1	(0.98)	1	(1.30)	1	(3.33)	0.537

*NRs* Non Responders, *ADs* Active Decliners, *NAs* Non-Attenders

The provided options ‘Not understanding the information booklet’ and ‘transport problems’ were not selected by anyone in either of the three groups

^a^Emerged from free text section

^*^The *p*-values are derived from Fisher’s exact test if not otherwise stated (^b^Chi-square test of Independence)

#### Combination of barriers

Pain and embarrassment was the most frequent combination of barriers to BSS (33.33%), followed by embarrassment and the enema (23.33%), and thirdly the combination of pain and worry about harm to the bowel (20.00%); (see Appendices 1 and 2 for details).

## Discussion

This study aimed to identify the demographic profile of BSS participants and non-participants, and importantly to compare profiles and reasons for BSS non-participation across three distinct subgroups of non-participants: those who do not respond to the screening invitation (NRs), those who actively decline the invitation (ADs) and those who confirm their appointment but then do not attend (NAs).

This is the largest study of self-reported participation in BSS to date, and the first to consider sub-groups of non-participants. In our study, as expected, the majority of questionnaire respondents had participated in BSS. However, within the non-participant group, variation allowed sub-group comparisons to be made; the majority were NRs, followed by ADs and then NAs. Compared to BSS participants, non-participants as a whole were more likely to be female, not married or living in a relationship, in paid employment, and from more socio-economically deprived backgrounds. These results closely match those described in a recent analysis of participation in the first 14 months of BSS roll-out [7]. Additionally, non-participants were less likely to state that they were interested in having the test when they first received the invitation and to have read the information leaflet that was sent as part of the invitation. This highlights a need to find other ways to engage invitees with the BSS opportunity.

We found important differences between the three sub-groups in their demographic profile and reported barriers to BSS participation. Specifically, NAs noted fewer barriers, and were more likely to report higher initial interest in having the BSS test, than the other two groups. The most frequently barriers reported by NAs focused on more practical elements of attendance (e.g. appointment problems and medical reasons), suggesting that this sub-group of non-participants may have had higher intentions to have the BSS test but that practical reasons prevented them from actually attending their appointment. This supports recent observations in non-attenders for diagnostic colonoscopy in the NHS Bowel Cancer Screening Programme [19]. In comparison, NRs and ADs were more likely to endorse anticipated pain and embarrassment as reasons for not participating in BSS, which suggests that these emotional barriers may have influenced the low initial interest in having the BSS test among these two sub-groups of non-participants.

Several of the barriers reported in this study have also been described in previous qualitative studies. The perceived lack of need has also been identified by Hall and colleagues [10], as part of their interview study of screeners and non-screeners; while, McCaffery and colleagues [11] also reported that perceived lack of susceptibility and lack of symptoms were important factors in the decision to decline screening. However, unlike the findings by McCaffery and colleagues [11] that suggested procedural barriers, such as anticipated embarrassment and pain and discomfort, to be reported as minor factors in the decision to decline, our study found these barriers to be much more prominent than previously reported.

The findings by Hall and colleagues [10] suggest that both BSS participants and non-participants anticipated the screening procedure to be unpleasant and invasive, and this often led to strong emotional responses to the invitation materials. In our study, perceiving the procedure to not be important but time consuming and embarrassing was prominent among those who did not read all the information materials. It is possible that with increasing awareness of the existing Bowel Cancer Screening Programme using gFOBt, and knowing that this test offers a much less invasive alternative at the age of 60, people might be more likely to question the invasive nature of BSS. Uptake of BSS might benefit from a better distinction between the two aspects of the programme, with bowel scope aiming to prevent CRC and the current gFOBt test being squarely aimed at detecting CRC early.

Distinguishing between different groups of BSS non-participants enables screening programme managers, practitioners, and policy makers to identify different barriers for different subgroups and develop more targeted interventions. Such interventions could include the use of narratives from previous participants, or reference to data showing that a vast majority of people who had the test report positive experiences. Similarly, previous research has identified that offering same-sex practitioners is a popular option, particularly among women who previously did not respond to the test invitation, and may be an important factor in helping to reduce anticipated embarrassment [20, 21]. A more proactive approach to informing invitees about the intensity of pain and discomfort to expect, and of ways these can be reduced, would also be important. For example, enabling screening invitees to choose early on whether they prefer pain relief in the form of Entonox, and when it is given (either in response to discomfort or pre-emptively to avoid pain and discomfort) could be considered.

Finally, while NAs represent a small proportion of BSS non-participants, they should not be forgotten. The programme has the strongest mandate to try and help these individuals realize their intention to have the test. For NAs, the main barriers were practical and medical aspects and so, for this group specifically, more autonomy over the appointment booking system and reminders of the self-referral process may be particularly important.

This study had a number of limitations. The study was carried out in primary care which enabled us identify eligible respondents without having to interfere with the invitation process. However, it should be acknowledged that we were limited to selecting among practices who were located in areas in which BSS had been rolled out. Despite our best efforts not all practices were available to participate so our selection of 28 practices did not constitute a probabilistic sample which may have introduced bias.

The survey had a response rate of only 45.8%, which is good compared with many similar CRC screening surveys, but survey respondents were more likely to come from affluent areas and be female which may well have caused uptake in this study to be significantly higher compared with what has been reported in the programme [7]. Overall, it is important to acknowledge that compared with the general population our sample was also more affluent and educated. In addition, as we were unable to provide translated versions of our questionnaires, our ability to identify barriers specific to different ethnic groups was limited.

Although our study analysed barriers of screening based on self-reported uptake, the results remain relevant as the self-reported screening behaviours proved to be very accurate. In our questionnaire, we asked responders to state whether they would allow the research team to check their screening status in their health records. While a total of 1232 responders gave permission to look up their participation records, only 888 could correctly be identified. This discrepancy may have been the result of unclear handwriting, as responders had to provide their name, date of birth and postcode by hand. Comparing the self-reported uptake of identified responders with participation recorded by the programme, shows accurate self-reported participation in 98.8% (*N* = 759) of responders. Similarly, 90.9% (*N* = 109) of those who declared that they did not participate had matching records. The lower accuracy for BSS non-participants is likely caused by them taking part after completing the questionnaire. Objective uptake data were obtained after study participants gave their permission and sent back the questionnaire to the research team. Relatedly, as a result of the relatively small proportion of BSS non-attenders, this study was not powered to properly test the significance levels of the comparison across barriers by SES or by non-participant subgroups.

A further limitation is that the study did not assess the delay between having received the BSS invitation and answering the questionnaire. Although the analysis was restricted to responders who indicated that their age was between 55 and 57 years at the time of the questionnaire, the length of time period between receiving BSS invitation and the questionnaire could have influenced the naming of the participation barriers.

Our study has strongly implicated pain and embarrassment of the test as common barriers. While this finding is a useful starting point, there would be value in further refining exactly what aspects of the test are perceived as painful and embarrassing, in order to develop potential strategies to make the test more acceptable. Furthermore, the fact that the combination of both terms was frequently mentioned, indicates that there might be some considerable overlap. Understanding the mechanisms behind this relationship might also lead to better decisions about how to intervene.

Most importantly, future research needs to identify how to balance the tension between not wanting to put people off and presenting enough information about the test to reassure those with strong negative preconceptions. In addition to written information, this may well include more facile, flexible and personal approaches, such as targeted messages, and patient navigation. Future research could also identify enabling factors by asking participants more directly what helped them overcome some of the barriers that were endorsed by them.

Not needing the test was another frequently mentioned barrier by NRs and ADs. Future research needs to identify the motivation behind citing this barrier, but it is likely that there is a lack of understanding of the unprecedented public health benefit associated with FS, particularly its ability to prevent CRC. The UK FS Trial went some way to develop pictorial information about the adenoma-cancer sequence, which was found to significantly increase knowledge and motivation [22]. Unfortunately, this approach has not been adopted in the education provided with the current information leaflet.

## Conclusions

This study highlighted that there are important variations between different types of non-participants in the bowel scope screening branch of the BCSP. Pain and embarrassment seem to be important concerns among those who either never respond or actively decline the offer. By contrast, people who initially confirm their invitation for bowel scope screening but do not attend their appointment are more interested and informed, and tend to come against more specific issues relating to acting on their intention. Interventions to improve uptake among these different types of non-participants should be more nuanced and use targeted strategies to improve uptake. In the short term, this should involve placing greater emphasis on the perceived benefits of the test as part of the initial invitation, using additional reminders for the test and the ability to self-refer, and more flexible appointment booking systems to reduce the number of NA’s.

## Appendix 1

**Table 6:** Most frequent combinations of reasons for not participating across the three non-participant groups (*N* = 120)

	Total (*N* = 120)			NRs (*N* = 66)			ADs (*N* = 43)			NAs (*N* = 11)			*p*-value^*^
	Rank	*N*	%	Rank	*N*	%	Rank	*N*	%	Rank	*N*	%	
Pain + embarrassing	1st	40	(33.33)	1st	26	(39.39)	1st	12	(27.91)	5th	2	(18.18)	0.295
Embarrassing + enema	2nd	28	(23.33)	2nd	17	(25.76)	2nd	9	(20.93)	5th	2	(18.18)	0.807
Pain + harm	3rd	24	(20.00)	4th	12	(18.18)	2nd	9	(20.93)	2nd	3	(27.27)	0.704
Pain + enema	4th	23	(19.17)	3rd	13	(19.70)	4th	7	(16.28)	2nd	3	(27.27)	0.653
Embarrassing + harm	5th	18	(15.00)	6th	10	(15.15)	4th	7	(16.28)	2nd	3	(27.27)	0.575
Embarrassing + not needed	6th	17	(14.17)	5th	11	(16.67)	7th	5	(11.63)	–	0	(0.00)	0.400
Medical reasons + other test	12th	11	(9.17)	32nd	2	(3.03)	7th	5	(11.63)	1st	4	(36.36)	0.003

*NRs* Non Responders, *ADs* Active Decliners, *NAs* Non-Attenders

^*^The *p*-values are derived from Fisher’s exact test

## Appendix 2

**Table 7:** Combination of reasons mentioned by all in percentages of occurrence (*N* = 120)

	Worried about pain	Embarrassing	Worried about harm	Not needed	Appointment problems	Enema	No time	Worried about results	Medical reasons	Unacceptable	Bad experience	Other test	Forgot	Financial problems
Worried about pain	–	33.33	20.00	14.17	6.67	19.17	5.83	6.67	0.83	7.50	4.17	0.83	0.83	0.83
Embarrassing		–	15.00	14.17	10.83	23.33	5.00	6.67	0.83	5.83	3.33	0.83	1.67	1.67
Worried about harm			–	13.33	6.67	9.17	3.33	5.83	0.83	4.17	2.50	0.83	0.00	0.00
Not needed				–	5.00	10.00	1.67	2.50	0.00	4.17	1.67	0.00	0.83	0.00
Appointment problems					–	5.83	13.33	3.33	0.00	3.33	0.83	0.00	2.50	0.83
Enema						–	1.67	3.33	0.83	4.17	4.17	0.00	1.67	0.83
No time							–	2.50	0.83	0.83	0.00	0.83	1.67	0.00
Worried about results								–	0.00	0.83	0.83	0.00	0.00	0.00
Medical reasons									–	0.00	0.00	9.17	0.83	0.00
Unacceptable										–	0.83	0.00	0.00	0.00
Bad experience											–	0.00	0.00	0.00
Other test												–	0.00	0.00
Forgot													–	0.00
Financial problems														–

## Abbreviations

AD: Active Decliner
BSS: Bowel Scope Screening
CRC: Colorectal cancer
FET: Fischer’s Exact Test
FS: Flexible Sigmoidoscopy
IMD: Index of Multiple Deprivation
NA: Non-attender
NR: Non-responder
UCL: University College London

## Footnotes

1 Due to low numbers we did not stratify combination of barriers by sub-group.
